# Failure to Re-identify and Manage Known Dementia Associated With Hospital Readmission and LOS: A Retrospective Cohort Study

**DOI:** 10.1177/07334648251368428

**Published:** 2025-08-26

**Authors:** Kara Cappetta, Luise Lago, Jan Potter, Lyn Phillipson

**Affiliations:** 1 Australian Health Services Research Institute [Closed Institution], 8691 University of Wollongong, NSW, Australia; 2 School of Health & Society, 8691 University of Wollongong, NSW, Australia; 3 Australasian Health Outcomes Consortium, 8691 University of Wollongong, NSW, Australia; 4 Centre for Health Research Illawarra Shoalhaven Population, [Closed Institution], 8691 University of Wollongong, NSW, Australia; 5 Illawarra Shoalhaven Local Health District, Wollongong, NSW, Australia

**Keywords:** clinical coding, hospital outcomes, dementia, health services research, longitudinal

## Abstract

The extent to which known dementia is re-identified and managed during hospitalization and the impact of this on patient outcomes warrant investigation. Among 7,919 patients with an index admission coded with dementia between 1 July 2006 to 30 June 2015 in a regional health district in Australia, we examined total length of stay (LOS) and Difference from Expected LOS (using mixed linear regression and quantile regression), and 30-day readmission (using a generalized linear mixed model). In models adjusted for age, complexity, specialty, and discharge destination, uncoded stays had significantly shorter total LOS (β = −2.9, 95% CI: −3.5, −2.3) and Difference from Expected LOS (β = −5.2, 95% CI: −5.8, −4.6). Quantile regression results confirmed these findings but were smaller in effect size. People without dementia coded were 1.2 times more likely to be re-readmitted (95% CI: 1.1, 1.3) than when coded. Consistent re-identification and active management of known dementia is needed to improve long-term patient and health system outcomes.

**What this study adds**

 • A unique contribution of this study is that it only includes people with a pre-existing hospital diagnosis of dementia and follows them over time. This research emphasizes the importance of not only initial dementia identification but also re-identification and management across all subsequent hospitalizations.
 • Among older adults hospitalized with a known dementia diagnosis, lack of re-identification and clinical management of dementia (using coding as a proxy) were associated with an increased risk of 30-day readmission and shorter than expected length of stay (LOS).
 • Treating specialty and discharge destination were also associated with LOS and likelihood of readmission.

**Application of study findings**

 • When a known dementia diagnosis is not identified and managed in hospital admissions, patients are more likely to experience short-term readmission.
 • There are points within a patient's care whereby dementia must be considered to improve management of inpatient clinical care—including at the outset and at point of discharge or transfer. The reidentification and active management of dementia is also impacted by the treating specialty.

## Introduction

People living with dementia who are admitted to hospital experience a change in environment, which can be disorienting and confusing, and result in poor clinical outcomes. Hospitalization can accelerate cognitive (Fogg et al., 2018; Krause et al., 2019) and functional decline (Timmons et al., 2016), and result in avoidable complications from conditions common amongst people with dementia (e.g., malnutrition and incontinence). Comorbid conditions may be unmanaged in hospital if care is focused on a patient’s primary diagnosis, or cognitive and behavioral symptoms (Kurrle et al., 2012). Consequently, older people hospitalized with cognitive impairment have an increased risk of poor health outcomes, such as mortality, admission to nursing home, increased length of stay (LOS) (Fogg et al., 2018), and readmission (Sakata et al., 2018).

Lack of identification and appropriate management of dementia in the hospital setting can increase the likelihood of adverse patient outcomes. Factors associated with increased LOS include type and severity of dementia (Zekry et al., 2008; Zilkens et al., 2009), presence of medical comorbidities (Zilkens et al., 2009; Zekry et al., 2008; King et al., 2006), sociodemographic characteristics (Zilkens et al., 2009), and other clinical factors, such as delirium, quality of life (Lang et al., 2010), and referral to an Aged Care Assessment Team (King et al., 2006). Assessment for cognition during acute admissions, however, is often inadequate (Timmons et al., 2016). This may result in sub-optimal decisions about access to specialist services, staffing levels, and discharge planning (Timmons et al., 2016).

There is conflicting evidence on whether people with dementia are at increased likelihood of readmission. Estimates of increased risk of readmission relative to people without dementia range from 3 to 13% in cohort studies. There is no evidence of increased risk in case–control studies (Pickens et al., 2017). This may be due in part to a focus on comparing readmission rates amongst patients with and without dementia, rather than when dementia is identified and actively managed across repeated hospitalizations. Medical coding of dementia decreases across subsequent admissions (Cappetta et al., 2020), which could contribute to inconsistent evidence.

The existence of under-coding (Cappetta et al., 2020; Organisation for Economic Co-operation and Development, 2017) and under-documentation (Chodosh et al., 2004; Crowther et al., 2016; Laurila et al., 2004) of dementia in the hospital setting is well established. Until July 2015, Australian clinical coding rules specified that conditions were only coded when significant in terms of treatment required, investigations needed, and resources used (Australian Consortium for Classification Development, 2017). Consequently, prior to July 2015, coding can be considered a proxy measure for active clinical management, with a lack of coded diagnosis indicating no active management in care.

Identifying characteristics that increase risk of poor outcomes can inform interventions to improve health system and patient outcomes by reducing readmission rates and LOS (Sakata et al., 2018). Retrospective longitudinal studies have found increasing age, low cognitive screening score, difficulties with daily activities, and high numbers of hospital admissions are associated with dementia being identified and coded (and thus managed) during a hospital admission (Sommerlad et al., 2018). To improve inpatient care, it is important to understand when dementia is not being identified and managed. The aim of this study was to estimate the association between identification and active management of dementia (using coding as a proxy) on LOS and risk of readmission following a first coded (index) admission with dementia.

## Methods

### Study Design

This study was a population-based retrospective cohort study of hospital utilization for patients following an index admission for dementia.

### Setting

The setting was a regional local health district of New South Wales (NSW), Australia, the Illawarra Shoalhaven Local Health District (ISLHD). ISLHD consists of a network of eight hospitals, including three acute care hospitals, servicing a population of over 400,000 residents (ISLHD, 2018). The study was approved by the Health and Medical Human Research Ethics Committee, University of Wollongong/Illawarra Shoalhaven Local Health District (2017/262).

### Participants

The study population consisted of residents of the ISLHD catchment region aged 65 years or over at first admission coded with dementia (i.e., index admission) who had at least one subsequent hospital admission during the study period. Exclusions were patients aged less than 65 years at index (i.e., early onset dementia), non-residents (to ensure long-term follow-up and representativeness), and people who died in hospital on their index hospitalization. Subsequent admissions for patients undergoing dialysis were also excluded (*n* = 7,567 episodes removed) as each separation was not considered to be an independent opportunity for assessment and diagnosis of dementia, and accounted for a large volume of separations. Patients with more than 45 admissions were excluded as outliers.

### Variables

The main study factor was coding of a diagnosis of dementia in the medical record during a post-index hospital stay. Uncoded dementia refers to a prior hospital diagnosis of dementia (i.e., previously coded dementia) that was not coded (i.e., actively managed) in the subsequent admission. Dementia was identified using International Statistical Classification of Diseases and Related Health Problems, Tenth Revision, Australian Modification (ICD-10-AM) Ninth Edition (ACCD, 2017) diagnosis codes of F00 (Dementia in Alzheimer’s disease), F01 (Vascular dementia), F02 (Dementia in other diseases classified elsewhere), F03 (Unspecified dementia), F05.1 (Delirium superimposed on dementia), and G30 (Alzheimer’s disease) (Australian Institute of Health and Welfare, 2012). For stays with multiple episodes, dementia was considered coded if any episode had dementia coded. A diagnosis of dementia in the coded medical record was used as a proxy for active clinical management, as per contemporaneous coding standards (ACCD, 2017).

The primary outcomes were total length of stay (LOS) and 30-day readmission. Total LOS was calculated by concatenating episodes into stays (accounting for change of care type) and calculating the difference between admission and separation date (same day episodes were given LOS of one). Readmission was defined as admission to any hospital in the ISLHD region within 30 days of the discharge date. Readmissions associated with the index admission were excluded, as dementia was always coded in the index admission by definition. The secondary outcome was Difference from Expected LOS, derived using the difference between the patients’ total LOS and the Australia-Refined Diagnosis Related Group (AR-DRG) benchmark (Lim et al., 2012; McCusker et al., 2003; Motzek et al., 2016). LOS benchmarks for AR-DRG v6.0x were identified using the Independent Hospital Pricing Authority (IHPA) Round 17 (2012–2013) Cost Report (IHPA, 2015).

Potential confounders of the association between dementia coding and LOS or readmission were identified in the dataset. These included sociodemographic characteristics (sex; age group: 65–74, 75–84, and 85+, country of birth; non-English-speaking background (NESB)); admission characteristics (treating specialty, discharge destination, dementia diagnosis type: principal diagnosis or additional diagnosis, and care type: acute, sub-acute, non-acute, or psychiatric); and comorbidities. Treating specialty accounted for multi-episode stays (flagged across all episodes). Comorbid conditions were hypertension (I10–I13 and I15), chronic lower respiratory disease (J40–J47), cerebrovascular disease with occlusion/stenosis (I63–I64, I66, G46, and I67.8), malignancy (C00–C95), chronic kidney disease (N18), hyperlipidemia (E78), diabetes mellitus (E10–E14), heart failure (I50), ischemic heart diseases (I20, I21, and I24–I25), arrhythmia (I46 and R00), and Parkinson’s disease (G20). Comorbidities were summarized using Charlson’s Comorbidity Index (CCI) (excluding dementia) (Sundarajan et al., 2004), with multi-episode stays recorded with the highest CCI score. Missing data was low (all data items <0.3%, except health insurance, 12.6%). Adjusted models used case-wise deletion.

### Data Sources

Unit-record data was extracted from the Admitted Patient data collection on the Illawarra Health Information Platform. This platform consists of a non-identifiable databank and a records linkage system for all patients attending ISLHD facilities since 2001 (Centre for Health Research Illawarra Shoalhaven Population, 2018). Data were extracted for the study period from 1 July 2006 to 30 June 2015 and a 5-year lookback period from 1 July 2001 to reduce the chance that a hospital diagnosis of dementia was identified prior to the study period.

### Statistical Analysis

Characteristics of the study cohort were described using proportions. The percentage of stays with dementia coded was measured. The effect of dementia coding on total LOS and Difference from Expected LOS was modeled using mixed linear regression, accounting for multiple hospitalizations per patient. The results of mixed logistic regression models were presented as unadjusted and adjusted odds ratios (OR and aORs), with adjustment for age group, NESB, CCI group, specialty, and discharge destination. These analyses were performed using SAS version 9.4 (SAS Institute Inc., Cary, NC).

A sensitivity analysis was carried out for total LOS and Difference from Expected LOS models using quantile regression with Gauss–Laguerre quadrature. This is a more flexible and robust approach to regression on the mean, which estimates the effect of the study factor on a range of percentiles of the distribution of the outcome variable (Koenker & Hallock, 2001). Estimates were produced for the 25^th^, 50^th^ (median), 75^th^, and 90^th^ percentile (Supplemental Table 1-4) and results reported for the median. Quantile regression models were implemented in RStudio with R version 4.1.3 using the lqmm function. All estimates include 95% confidence intervals (CIs), and statistical tests were assessed at the 5% level of significance. The quality of the fit of each model was described using Akaike Information Criterion.

## Results

There were 7,919 people with an index admission with dementia coded in the study period. A total of 34,766 subsequent inpatient episodes were observed, concatenated into 23,838 hospital stays.

### Patient and Admission Characteristics

The study cohort was 56.7% female, with 73.0% aged 80 years or over (Table 1). The index admission was predominantly acute (89.6%), and dementia was mostly coded as an additional diagnosis (91.2%). Dementia type was unspecified in over half (55.4%) of index admissions, with Alzheimer’s disease the most common specified type. Most patients had a mild (38.7%) or moderate/severe CCI (29.0%), and the most common comorbidities were urinary tract infection (49.4%) and hypertension (49.2%).

**Table 1.** Study Cohort Characteristics at Index Admission

	Study cohort
	*n* (%)
Sex	
Female	4,490 (56.7)
Male	3,429 (43.3)
Age (years)	
65–69	283 (3.6)
70–74	574 (7.3)
75–79	1,271 (16.1)
80–84	2,110 (26.6)
85–89	2,292 (28.9)
90+	1,389 (17.5)
Aboriginal &/or Torres Strait Islander People^a^	
No	7,857 (99.2)
Yes	50 (0.6)
Country of birth^a^	
Australia	4,977 (62.9)
Other English speaking	1,149 (14.5)
Non-English-speaking background	1,793 (22.6)
Health insurance on admission^a^	
No	5,116 (64.6)
Yes	1,809 (22.8)
Comorbidities^b^	
Urinary tract infection	3,916 (49.4)
Hypertension	3,900 (49.2)
Delirium	3,112 (39.3)
Tendency to fall	2,977 (37.6)
Diabetes	2,100 (26.5)
Pneumonia	1,862 (23.5)
Heart failure	1,728 (21.8)
Ischemic heart disease	1,532 (19.3)
Syncope/collapse	1,439 (18.2)
Chronic kidney disease	1,328 (16.8)
CCI group^b^	
0	2,554 (32.3)
1, 2 (mild)	3,066 (38.7)
≥3 (moderate/severe)	2,300 (29.0)
Dementia diagnosis type	
Principal diagnosis	695 (8.8)
Additional diagnosis	7,224 (91.2)
Dementia type^a^	
Unspecified dementia	4,379 (55.4)
Alzheimer’s disease^c^	1,544 (19.6)
Delirium superimposed on dementia	1,033 (13.1)
Vascular dementia	586 (7.4)
Dementia in other diseases classified elsewhere	363 (4.6)
Care type^a^	
Acute	7,094 (89.6)
Sub- and non-acute	724 (9.1)
Psychiatric	101 (1.3)

*Note.* Study cohort, *n* = 7,919. CCI—Charlson Comorbidity Index.

^a^Proportion of missing, unknown, other, or not stated: Aboriginal/Torres Strait Islander, 0.2%. Country of birth, 0%. Health insurance on admission, 12.6%. Patient type, 0%. Dementia type, 0.2%.

^b^Comorbidities across all admissions, with 5-year lookback period (principal and/or additional diagnosis).

^c^Alzheimer’s disease includes Dementia in Alzheimer’s disease (F00) and Alzheimer’s disease (G30).

### Dementia Coding Rates

Dementia was coded (identified and managed) in 60.1% of total stays, including in just over half of stays for females (59.6%) and males (60.8%) (Table 2). Coding increased with age, from 55.2% of stays for patients aged 65 to 74 to 61.5% of stays for patients aged 85 and over. The rate of dementia coding (identification and management) varied across specialty, with the highest rate for patients treated by a geriatrician (80.6%) and the lowest for patients treated by emergency medicine (44.6%). Patients with no comorbidities (CCI = 0) had dementia coded (identified and managed) more frequently (68.4%) than medically complex patients (CCI group 1 or more). Patients discharged to a nursing home had dementia coded (identified and managed) more frequently (79.6% of stays) compared to other discharge destinations.

**Table 2.** Dementia Coding: By Sociodemographic and Clinical Characteristics (Stay Level)

	Total stays (*n*)	Stays coded with dementia (%)
Sex		
Female	13,023	59.6
Male	10,815	60.8
Age		
65–74	2,477	55.2
75–84	10,130	59.8
85+	11,231	61.5
Non-English-speaking background		
Yes	5,902	62.2
No	17,936	59.4
CCI group		
0	5,296	68.4
1, 2 (mild)	9,685	63.5
≥3 (moderate/severe)	8,857	51.5
Specialty^a^		
Geriatrics	6,018	80.6
Emergency medicine	5,311	44.6
General medicine	4,326	61.9
Discharge destination^b^		
Home	13,697	42.4
Nursing home	2,415	79.6
Other hospital	5,407	64.3
Total stays	23,838	60.1

*Note.* CCI – Charlson Comorbidity Index.

^a^Other specialty, or not stated: *N* = 8,183.

^b^Other discharge destination (including type change), or not stated: *N* = 2,319.

### Length of Stay

Uncoded (unidentified and unmanaged) dementia was associated with a reduced total LOS (β = −7.4, 95% CI: −8.1, −6.8) than when dementia was coded (identified and managed) (Table 3). There was also a shorter LOS for uncoded stays for Difference from Expected LOS (β = −5.2, 95% CI: −5.8, −4.6). The finding of shorter LOS for uncoded stays was confirmed in models for median total LOS (β = −7.8, 95% CI: −8.9, −6.7) and median Difference from Expected LOS (β = −1.2, 95% CI: −1.7, −0.7) (Table 3). After adjustment for age group, NESB, CCI, specialty, and discharge destination, uncoded (unidentified and unmanaged) dementia was still associated with a shorter LOS (Table 4); however, the effect size was smaller (e.g., mean total LOS: β = −2.9, 95% CI: −3.5, −2.3; median total LOS: β = −1.6, 95% CI: −2.0, −1.2).

**Table 3.** Unadjusted Regression Models for Total Length of Stay (LOS), Difference from Expected LOS, and Readmission (Person-Level Clustering)

	Total LOS^a^		Difference from expected LOS^b^		Readmission^c^
Variable	*Mean regression* Estimate (95% CI)	*Quantile regression* Estimate (95% CI)	*Mean regression* Estimate (95% CI)	*Quantile regression* Estimate (95% CI)	*Logistic regression* OR (95% CI)
Uncoded dementia	−7.4 (−8.1, −6.8) ***	−7.8 (−8.9, −6.7) ***	−5.2 (−5.8, −4.6) ***	−1.2 (−1.7, −0.7) ***	1.7 (1.6, 1.8) ***

*Note.* LOS—length of stay; CI—confidence interval; OR—odds ratio; ***p < 0.001.

^a^*N* = 23,838. Model fit: Mean regression, Akaike Information Criterion (AIC) = 221,296; Quantile regression, AIC = 197,427.

^b^*N* = 23,838. Model fit: Mean regression, AIC = 220,103; Quantile regression, AIC = 194,856.

^c^*N* = 23,838. Model fit: AIC = 22,168.

**Table 4.** Adjusted Regression Models for Total Length of Stay (LOS), Difference from Expected LOS, and Readmission (Person-Level Clustering)

	Total LOS^a^		Difference from expected LOS^b^		Readmission^c^
Variable	*Mean regression* Estimate (95% CI)	*Quantile regression* Estimate (95% CI)	*Mean regression* Estimate (95% CI)	*Quantile regression* Estimate (95% CI)	*Logistic regression* aOR (95% CI)
Uncoded dementia	−2.9 (−3.5, −2.3)***	−1.6 (−2.0, −1.2)***	−0.9 (−1.5, −0.3)**	−0.2 (−0.3, −0.1)**	1.2 (1.1, 1.3)***
Female	−0.2 (−0.8, 0.4)	0.0 (−0.1, 0.1)	−0.2 (−0.8, 0.4)	0.1 (−0.0, 0.3)	1.0 (0.9, 1.0)
Age group					
65–74 (ref)	-	-	-	-	-
75–84	−1.1 (−2.1, −0.1)	−3.1 (−4.6, −1.5)***	−1.0 (−2.0, −0.1)*	−0.2 (−0.5, 0.1)	0.8 (0.7, 0.9)***
85+	−2.8 (−3.8, −1.8)***	−3.1 (−4.6, −1.5)***	−2.7 (−3.7, −1.8)***	−0.4 (−0.6, −0.1)*	0.7 (0.6, 0.8)***
Non-English speaking	2.5 (1.9, 3.2)***	0.1 (−1.6, 0.5)	2.4 (1.7, 3.0)***	0.1 (−0.1, 0.3)	1.0 (0.9, 1.1)
CCI group					
0 (ref)	-	-	-	-	-
1,2 (mild)	0.1 (−0.7, 0.9)	−0.2 (−0.5, 0.2)	0.0 (−0.7, 0.8)	−0.1 (−0.3, 0.1)	1.2 (1.1, 1.4)***
≥3 (moderate/severe)	0.0 (−0.8, 0.8)	−0.0 (−0.1, 0.1)	−0.5 (−1.3, 0.3)	0.1 (−0.1, 0.3)	1.3 (1.2, 1.5)***
Specialty					
General medicine (ref)	-	-	-	-	-
Emergency medicine	−7.7 (−8.4, −7.0)***	−4.3 (−4.7, −3.8)***	−6.1 (−6.8, −5.3)***	−1.1 (−1.2, −0.9)***	1.5 (1.4, 1.6)***
Geriatrics	12.2 (11.5, 12.9) ***	10.3 (9.5, 11.0)***	11.8 (11.1, 12.5)***	10.0 (9.4, 10.6)***	0.3 (0.2, 0.3)***
Discharge destination					
Home (ref)	-	-	-	-	-
Nursing home	0.1 (−1.0, 1.1)	0.5 (−0.4, 1.4)	0.9 (−0.1, 1.9)	1.7 (1.1, 2.2)***	0.8 (0.7, 0.9)**
Other hospital	20.4 (19.7, 21.1)***	17.7 (16.6, 18.9)***	20.3 (19.6, 20.9)***	16.5 (15.5, 17.4)***	0.7 (0.6, 0.8)***

*Note.* LOS—length of stay; aOR—adjusted odds ratio; CCI—Charlson Comorbidity Index; CI—confidence interval. Significance levels: ***p < 0.001; **p < 0.01; *p < 0.05; ref = Reference category.

^a^*N* = 23,838. Model fit: Mean regression, Akaike information criterion (AIC) = 216,229; *Quantile* regression, AIC = 192,398.

^b^*N* = 23,838. Model fit: Mean regression, AIC = 215,240; *Quantile* regression, AIC = 190,427.

^c^*N* = 23,838. Model fit: Logistic regression, AIC = 21,050.

Among all admissions (coded and uncoded), patients admitted under an emergency specialist (Table 4) had a 4.3 days shorter median total LOS (β = −4.3, 95% CI: −4.7, −3.8) compared to those admitted under general medicine. This result was consistent for Difference from Expected LOS but a smaller size (β = −1.1, 95% CI: −1.2, −0.9). Patients admitted under a geriatrician had a longer median total LOS (β = 10.3, 95% CI: 9.5, 11.0) compared to those admitted under general medicine. This result was consistent for Difference from Expected LOS (β = 10.0, 95% CI: 9.4, 10.6). Discharge to other hospital was also associated with increased LOS (median regression estimate for Difference from Expected LOS: β = 16.5, 95% CI: 15.5, 17.4).

### Readmission

Uncoded (unidentified and unmanaged) dementia was associated with 1.7 times the risk of 30-day readmission compared to admissions where dementia was coded (identified and managed) (OR = 1.7; 95% CI: 1.6, 1.8) (Table 3). After controlling for age group, NESB, CCI, specialty, and discharge destination (Table 4), there remained 1.2 times the risk of readmission (adjusted OR (aOR) = 1.2, 95% CI: 1.1, 1.3). Risk of readmission was higher for patients admitted under emergency admission in the previous episode (aOR = 1.5, 95% CI: 1.4, 1.6) and lower when seen by a geriatrician (aOR = 0.3, 95% CI: 0.2, 0.3) compared to a general medicine specialist.

## Discussion

To our knowledge, this study is the first to evaluate the impact of re-identification and active clinical management of dementia on admitted patient outcomes in a cohort with known dementia. Coding of dementia was used as a proxy for identification and management and was associated with both LOS and risk of readmission among people with dementia.

### Impact of Coding on LOS

Reduction of LOS is a key hospital focus as unnecessary days in hospital can result in increased costs and increased hospital-acquired patient complications (Rojas-Garcia et al., 2017). Health systems also use LOS as a proxy for hospital efficiency (Siddique et al., 2021). For people living with dementia, reduced LOS is especially important, as they can decline when moved out of familiar settings (Porock et al., 2013) and are at a risk of hospital-acquired infection or delirium with longer LOS (Fogg et al., 2018).

Shorter LOS, however, may lead to a false economy of patients being discharged earlier only to be readmitted due to a lack of management of their diagnosis in the previous admission. Our study found that patients whose dementia was unmanaged had shorter LOS; however, their likelihood of readmission increased. This suggests that lack of identification and management of dementia during an admission sets patients on a trajectory that puts them at risk of poor outcomes. Identification of dementia during an admission is therefore vital to be aware of the patient’s greater susceptibility to adverse events in hospital and to prompt preventative action (Fogg et al., 2018) even if this results in additional care and a longer stay. Our findings highlight the potential trade-off between decreased LOS and increased risk of readmission, a particularly undesirable outcome for people living with dementia. Reducing LOS may also shift costs of care to the outpatient setting (Rojas-Garcia et al., 2017).

Findings from the present study must be interpreted in the context of the treating specialty. The rate of identification and active management (coding) of dementia varied across specialty, with geriatrics recording the highest (80.6%) and emergency medicine recording the lowest (44.6%). Patients treated by an emergency specialist had lower LOS, while those treated by a geriatric specialist remained in hospital the longest. This implies that more comprehensive management is being provided to patients with dementia when treated by the geriatric specialty—whereby they are more likely to have their dementia identified and appropriately managed, and have a significantly reduced likelihood of short-term readmission. Conversely, patients discharged under the emergency medicine specialty may be more likely to have had only their presenting symptoms managed without further assessment of contributing factors. This in turn may result in a lack of treatment of ongoing conditions, such as delirium and dementia, leading to the increased risk of readmission demonstrated in the present study. A clinical implication is that hospitals, particularly the Emergency Department (ED), may benefit from a dedicated geriatric service to provide more targeted care to patients with dementia. For instance, an ED intervention utilized advanced practice ED nurses with gerontological training to provide targeted geriatric assessment and streamlining of care in frail older adults (Wallis et al., 2018). The intervention resulted in reduced ED LOS, hospital admission and, if admitted, hospital LOS. A similar intervention implemented within the local health district and targeting cognitive impairment may improve dementia identification and mitigate some of the adverse outcomes experienced by hospitalized people with dementia.

Patients transferred to another hospital had an increased LOS. Approximately 1 in 3 (29%) hospitalizations of people with dementia in Australia in 2016–17 ended with the patient continuing care in hospital—via either transfer or a change in hospital care type (Australian Institute of Health and Welfare, 2019). This increased LOS may be due to delays in discharge planning or difficulties in organizing long-term care placement (Fogg et al., 2018; Richardson et al., 2019). The implication is that discharge and/or transfer is another point at which management of dementia can (and should) occur. Multi-component pre- and post-discharge strategies that are focused around “bridging” interventions and a dedicated clinician responsible for transitional care can be employed to facilitate a better care experience and safer transitions (Rennke & Ranji, 2014; Richardson et al., 2019) for people with dementia. Failure to identify and manage dementia means a missed opportunity to manage transitions appropriately.

### Impact of Re-Identification and Management on Readmission

In this study, risk of readmission increased following an admission in which dementia was not identified or managed (not coded). Briggs et al. (2016) found that patients with dementia had increased risk of further admission or death within 12 months following an acute presentation. Our findings indicate that this risk may be increased when a patient’s dementia is not identified and appropriately managed in the previous admission. Patients thus may not receive the care they need (in and out of hospital) and subsequently return to hospital within 30 days for a potentially preventable readmission.

Consequences of unnecessary readmission for people with dementia include deteriorating health and increased risk of intensive care unit admission and death (Anderson et al., 2022; Fogg et al., 2018). Various strategies help to reduce preventable readmission; however, these tend to focus on home-based care by interdisciplinary teams and support to family carers (Ma et al., 2019). Our study highlights a novel factor not previously considered in hospital-based readmission interventions—the impact of identification and management of dementia during the previous admission. This supports other limited evidence that early identification of patients at risk for readmission is a critical first step to effectively reduce readmission in people with dementia (Ma et al., 2019) and further emphasizes the trade-off between reducing LOS at the expense of preventable readmission.

When dementia is not identified and managed during hospitalization, it sets patients on a trajectory that makes them more likely to be readmitted within 30 days. While timely identification of dementia upon hospital presentation is preferable (Department of Health and Aged Care, 2015), we have established the importance of attention to dementia, not solely at the index admission but at each hospitalization. Additional areas within the patient’s care trajectory where there is an opportunity to consider identification and management of dementia and concentrate efforts for intervention include across specific specialty areas (i.e., emergency medicine), and at the point of discharge or transfer to another hospital (i.e., transitional care). Further research into interventions focusing on these target areas would be beneficial to improve the identification and management of dementia in the hospital setting, and consequently, to reduce potentially avoidable readmissions.

### Need for Improved Identification and Management Across Hospitalizations

There are obstacles and complexities associated with establishing a hospital diagnosis of dementia, including time constraints, clinician hesitation, inadequate training, and acute causes of cognitive decline (Russ et al., 2012). While hospital-based interventions and models of care exist to support the identification and management of people with dementia, they often rely on screening (Agency for Clinical Innovation, 2017; MacDermott et al., 2017) and carer input (Clinical Excellence Commission, 2014) and can be time- and resource-heavy. A unique contribution of this study is that it only includes people with pre-existing hospital diagnoses of dementia. For this cohort, the process of identification should be more streamlined as the diagnostic information is available within the medical record. Our findings indicate that identification and management of dementia is also an issue amongst people with an established hospital diagnosis. This extends the issue of “one-off” identification, highlighting the importance of hospital staff seeing patients as being on a trajectory and needing to link their experience through subsequent admissions.

One option to improve identification and management in the hospital setting is to utilize existing documentation from medical records through automated systems. There is an opportunity to update centralized records systems to flag a patient’s diagnosis of dementia. Within NSW, this could be accomplished through the Electronic Medical Record (eMR) (NSW Health, 2014), which allows staff to document patient progress and test results electronically. The eMR includes a Patient Summary page that provides a “snap-shot” of relevant clinical information, with the ability to flag alerts relevant to the patient’s care. Well-designed computerized reminders and alerts can support clinical workflow and improve quality of care and patient safety (Bates et al., 2001; Davidson et al., 2004; Olakotan & Yusof, 2021). Using eMR to support the identification of an existing dementia diagnosis through a flag may assist with alerting the care team of a patient’s diagnosis. With this knowledge, the team could take measures to not only treat the presenting condition but also consider the patient’s needs more holistically, for instance, implementing hospital readmission risk interventions in hospital rather than post-discharge—thereby providing appropriate supports during admission that may prevent readmission in the future.

### Study Limitations

This study assumed that if dementia was coded it was actively managed, based on the relevant Australian Coding Standards (National Centre for Classification in Health, 2007). The diagnosis may have been managed, but not recorded on the medical record, or management not adequately described. Since this study, a change in coding rules required chronic conditions to be coded regardless of whether they were managed, and therefore did not meet criteria for clinical coding under ACS 0002 *Additional diagnoses* (ACCD, 2017). Additional research would be beneficial to understand the implications of under-coding following this change; however, active management may still be difficult to assess. The authors undertook a medical record audit to evaluate the coding change (forthcoming). Further limitations include known issues relating to the use of administrative data (Cummings et al., 2011) and the inability to adjust for potential confounding by unobserved factors, such as dementia severity, behavioral problems, living situation, and presence of a carer. Strengths of the study include the cohort size and ability to link data longitudinally and follow patients over time with a unique patient identifier. Outcomes were also assessed across multiple sites, which allowed for tracking of patients across hospitalizations and assessment of risk factors across different types of hospitals.

### Conclusion

Lack of active clinical management of dementia was found to increase the risk of readmission within 30 days and was associated with shorter LOS. Medical specialty was also found to be associated with LOS and readmission. These findings highlight multiple opportunities for identification and management of dementia across specific specialty areas (i.e., emergency medicine) and time points (i.e., upon discharge or transfer to another hospital). Recognizing that timely identification and adequate management of dementia during an admission prevents short-term readmission benefits all—the patient, clinicians (e.g., workload), and the hospital overall (e.g., costs). This research highlights the need for continuity of shared information across care episodes and settings across the dementia trajectory.

## Supplemental Material

Supplemental Material - Failure to Re-identify and Manage Known Dementia Associated With Hospital Readmission and LOS: A Retrospective Cohort Study Supplemental Material for Failure to Re-identify and Manage Known Dementia Associated With Hospital Readmission and LOS: A Retrospective Cohort Study by Kara Cappetta, Luise Lago, Jan Potter, and Lyn Phillipson in Journal of Applied Gerontology.
