# Supplementary material for: Failure to Re-identify and Manage Known Dementia Associated With Hospital Readmission and LOS: A Retrospective Cohort Study
Source: J Appl Gerontol. 2025 Aug 26;45(7):1360–9. doi: 10.1177/07334648251368428 (PMC13260741; doi:10.1177/07334648251368428)
Supplement: Supplemental Material - Failure to Re-identify and Manage Known Dementia Associated With Hospital Readmission and LOS: A Retrospective Cohort Study [file sj-pdf-1-jag-10.1177_07334648251368428.pdf]

SUPPLEMENTARY TABLES

Supplementary Table S1 Unadjusted quantile (25%, 50%, 75%, 90%) regression models for Total Length of Stay (LOS) (person-level clustering)

	Total LOS			
Variable	*25% Quantile regression* [a] Estimate (95% CI)	*50% Quantile regression* [b] Estimate (95% CI)	*75% Quantile regression* [c] Estimate (95% CI)	*90% Quantile regression* [d] Estimate (95% CI)
Un-coded dementia	-3.9 (-4.5, 3.3) ***	-7.8 (-8.9, -6.7) ***	-8.7 (-9.9, -7.5) ***	-11.6 (-13.8, -9.4) ***

Note: LOS – Length of Stay; CI – Confidence Interval; ***p<0.001.

(a) N=23,838. Model fit: 25% Quantile regression, Akaike information criterion (AIC)=187,190.
(b) N=23,838. Model fit: 50% Quantile regression, AIC=197,427.
(c) N=23,838. Model fit: 75% Quantile regression, AIC=207,338.
(d) N=23,838. Model fit: 85% Quantile regression, AIC=216,402.

Supplementary Table S2 Adjusted quantile (25%, 50%, 75%, 90%) regression models for Total Length of Stay (LOS) (person-level clustering)

	Total LOS [a]			
Variable	*25% Quantile regression* [a] Estimate (95% CI)	*50% Quantile regression* [b] Estimate (95% CI)	*75% Quantile Regression* [c] Estimate (95% CI)	*90% Quantile regression* [d] Estimate (95% CI)
Female	-1.4 (-3.5, 0.0)	2.3 (-2.0, 0.0)	3.9 (-1.3, 0.1)	-2.2 (-5.8, 1.5)
Age				
65-74 (ref)	-	-	-	-
75-84	-3.1 (-4.4, -1.7) ***	-3.0 (-4.6, -1.5) ***	-1.5 (-3.1, 0.2)	-1.0 (-5.1, 3.2)
85+	-3.1 (-4.4, -1.7) ***	-3.1 (-4.6, -1.5) ***	-1.5 (-3.1, 0.2)	-2.0 (-5.6, 1.7)
Non-English speaking	0.0 (-0.0, 0.0)	0.1 (-1.6, 0.5)	0.8 (-2.8, 1.8)	1.3 (-1.2, 3.8)
CCI				
CCI=0 (ref)	-	-	-	-
CCI=1,2	-0.6 (-1.2, 0.0) *	-0.2 (-0.5, 0.2)	0.2 (-3.9, 0.8)	-0.3 (-3.8, 3.1)
CCI≥3	-0.6 (-1.9, 0.0) *	-0.0 (-0.1, 0.1)	0.2 (-0.5, 0.8)	0.2 (-2.6, 2.9)
Un-coded dementia	-2. (-2.4, -1.6) ***	-1.6 (-2.0, -1.2) ***	-2.4 (-3.2, -1.6) ***	-6.7 (-10.8, -2.6) **
Specialty				
General medicine (ref)	-	-	-	-
Emergency medicine	-2.0 (-2.1, -1.9) ***	-4.3 (-4.7, -3.8) ***	-5.9 (-6.5, -4.6) ***	-8.4 (-11.5, -5.3) ***
Geriatrics	6.1 (5.7, 6.6) ***	10.3 (9.5, 11.0) ***	11.8 (10.5, 13.2) ***	7.1 (2.4, 11.7) **
Discharge destination				
Home (ref)	-	-	-	-
Nursing home	-1.6 (-0.8, 0.4)	0.5 (-0.4, 1.4)	-0.1 (-0.9, 0.7)	-2.5 (-4.9, -0.0) *
Other hospital	10.5 (9.4, 11.6) ***	17.7 (16.6, 18.9) ***	21.2 (20.3, 22.8) ***	17.4 (13.0, 21.7) ***

Note: LOS – Length of Stay; CCI – Charlson Comorbidity Index; CI – Confidence Interval; Significance levels: ***p<0.001; **p<0.01; *p<0.05; ref = Reference category.

(a) N=23,838. Model fit: 25% Quantile regression, Akaike information criterion (AIC) =183,059.
(b) N=23,838. Model fit: 50% Quantile regression, AIC = 192,398.
(c) N=23,838. Model fit: 75% Quantile regression, AIC =201,916.
(d) N=23,838. Model fit: 90% Quantile regression, AIC =211,493.

Supplementary Table S3 Unadjusted mean and quantile (25%, 50%, 75%, 90%) regression models for Difference in Expected LOS (person-level clustering)

	Difference from Expected LOS			
Variable	*25% Quantile regression* [a] Estimate (95% CI)	*50% Quantile regression* [b] Estimate (95% CI)	*75% Quantile regression* [c] Estimate (95% CI)	*90% Quantile regression* [d] Estimate (95% CI)
Un-coded dementia		-1.2 (-1.7, -0.7) ***	-3.4 (-6.7, -0.1)	-8.1 (-9.9, -6.3) ***

Note: LOS – Length of Stay; CI – Confidence Interval; ***p<0.001.

(a) Model did not converge.
(b) N=23,838. Model fit: 50% Quantile regression, AIC=197,427.
(c) N=23,838. Model fit: 75% Quantile regression, AIC=207,346.
(d) N=23,838. Model fit: 90% Quantile regression, AIC=214,232.

Supplementary Table S4 Adjusted mean and quantile (25%, 50%, 75%) regression models for Difference in LOS from Benchmark (person-level clustering)

	Difference from Expected LOS			
Variable	*25% Quantile regression* [a] Estimate (95% CI)	*50% Quantile regression* [b] Estimate (95% CI)	*75% Quantile regression* [c] Estimate (95% CI)	*90% Quantile regression* [c] Estimate (95% CI)
Female	0.0 (-0.2, 0.2)	0.12 (-0.02, 0.26)		
Age				
65-74 (ref)		-	-	-
75-84	-0.2 (-0.6, 0.1)	-0.2 (-0.5, 0.1)		
85+	-0.3 (-1.0, -0.3) ***	-0.4 (-0.6, -0.1) *		
Non-English speaking	0.1 (-0.2, 0.4)	0.1 (-0.1, 0.3)		
CCI				
0 (ref)		-	-	-
1,2	-0.1 (-0.3, 0.2)	-0.1 (-0.3, 0.1)		
3+	-0.1 (-0.4, 0.2)	0.1 (-0.1, 0.3)		
Un-coded dementia	1.2 (1.0, 1.4) ***	-0.2 (-0.3, -0.1) **		
Specialty				
General medicine (ref)		-	-	-
Emergency medicine	-0.9 (-1.1, -0.7) ***	-1.1 (-1.2, -0.9) ***		
Geriatrics	5.2 (4.7, 5.6) ***	10.0 (9.4, 10.6) ***		
Discharge destination				
Home (ref)		-	-	-
Nursing home	0.5 (0.1, 0.9) *	1.7 (1.1, 2.2) ***		
Other hospital	7.3 (6.9, 7.8) ***	16.5 (15.5, 17.4) ***		

Note: LOS – Length of Stay; CCI – Charlson Comorbidity Index; CI – Confidence Interval; Significance levels: ***p<0.001; **p<0.01; *p<0.05; ref = Reference category.

(a) N=23,838. Model fit: 25% Quantile regression, AIC=182,190.
(b) N=23,838. Model fit: 50% Quantile regression, AIC=190,427.
(c) Models did not converge.
